# Accessing Biospecimens from the H3Africa Consortium

**DOI:** 10.1089/bio.2017.0008

**Published:** 2017-04-01

**Authors:** Christine M. Beiswanger, Alash'le Abimiku, Nadia Carstens, Alan Christoffels, Jantina de Vries, Audrey Duncanson, Morne du Plessis, Maria Giovanni, Katherine Littler, Nicola Mulder, Jennifer Troyer, Louise Wideroff

**Affiliations:** ^1^ Genomic and Custom Services, Coriell Institute for Medical Research, Camden, New Jersey.; ^2^ IHVN H3Africa Biorepository, Institute of Human Virology-Nigeria, Abuja, Nigeria.; ^3^ International Research Center of Excellence, Institute of Human Virology-Nigeria, Abuja, Nigeria.; ^4^ Department of Epidemiology, Institute of Human Virology, University of Maryland School of Medicine, Baltimore, Maryland.; ^5^ Sydney Brenner Institute for Molecular Bioscience, University of the Witwatersrand, Johannesburg, South Africa.; ^6^ MRC Bioinformatics Unit, South African National Bioinformatics Institute, University of Western Cape, Bellville, South Africa.; ^7^ Bioethics at Department of Medicine, Faculty of Health Sciences, University of Cape Town, Cape Town, South Africa.; ^8^ Molecular and Physiological Science, Wellcome Trust, London, United Kingdom.; ^9^ Division of Human Genetics, Department of Pathology, University of Cape Town, Cape Town, South Africa.; ^10^ Microbial Genomics and Advanced Technologies, National Institute of Allergy and Infectious Disease, Rockville, Maryland.; ^11^ Wellcome Trust, London, United Kingdom.; ^12^ Computational Biology Group, Institute of Infectious Disease and Molecular Medicine, University of Cape Town, Cape Town, South Africa.; ^13^ Division of Genomic Sciences, National Human Genome Research Institute, Rockville, Maryland.; ^14^ Epidemiology and Clinical Studies, National Eye Institute, Bethesda, Maryland.

A pan-African consortium, Human Health and Heredity in Africa (H3Africa), was initiated in 2010 to build research programs and infrastructure in genomic medicine on the African continent.^1^ A key component of this initiative is making biospecimens and data collected during the initial H3Africa research studies available to external investigators for future research. Although policies and procedures for sharing H3Africa genomic data and biospecimens have been combined, this article will address the process developed to enable the ethical and efficient sharing of the biospecimens and associated data. To this end, biorepositories have been established in Nigeria (HVN H3Africa Biorepository [I-HAB]), Uganda (Integrated Biorepository of H3Africa Uganda [IBRH3AU]), and South Africa (H3Africa Biorepository at Clinical Laboratory Services, University of the Witwatersrand). The biorepositories will preserve the consortium's DNA and other sample collections and also facilitate their distribution to support additional research efforts by African scientists and eventually the global biomedical community.^2^ Each H3Africa principal investigator (PI) has agreed to donate a portion of the DNA collected in his/her H3Africa study while retaining the rest for his/her own future research. All biospecimens to be made available for distribution from the H3Africa biorepositories were collected under appropriate informed consent with institutional and regional or national ethical review.

Consortium working groups were established to develop polices for biospecimen sharing and access that respect the interests of all stakeholders. H3Africa stakeholders include the biospecimen donors and their community; the PI and his/her team collecting the specimens and primary data; the institutional, local, and regional ethics committees; the local or national government where samples were collected and stored; the scientists who are granted access to shared biospecimens for additional research; and the eventual consumers of biomedical knowledge to be gained from these studies.^3^ Fundamental concepts were drawn from existing biorepository best practice documents,^4–6^ which were used to inform the policies developed by investigators and biorepository experts across the H3Africa Consortium.

Key Elements of the H3Africa Biospecimen Sharing Policy

• Samples deposited in the H3Africa biorepositories are not owned by the biorepositories, but rather retained under the custodianship of the H3Africa biorepositories until they are approved for future research by the Data and Biospecimen Access Committee (DBAC).
• The Data and Biospecimen Access Committee must approve all requests to access the H3Africa biospecimens.
• The DBAC may consult donor PIs regarding caveats in appropriate use of the donated collections.
• Researchers on the African continent or who collaborate with African scientists will have priority access to samples.
• An embargo period for access to the biospecimens by outside investigators will allow time for publication of the H3Africa study for which the samples were initially collected;
• Commercialization of the biospecimens themselves is prohibited.

The H3Africa biorepositories will adhere to international standards for the implementation of standard operating procedures related to biospecimen processing, storage, shipping, tracking, and quality assurance/quality control. A set of minimum essential data elements determined by the H3Africa working groups will accompany each sample from the central collection laboratory to the H3Africa biorepositories for future shared distribution. Additional phenotypic, clinical, and genomic data for each sample will be available from the European Genome-phenome Archive (www.ebi.ac.uk/ega/home) through H3Africa-approved requests.^7^ An interactive searchable catalog is under development by H3ABioNet (the informatics resource of H3Africa) to allow public viewing of available biospecimen collections.

**FIG. 1.**
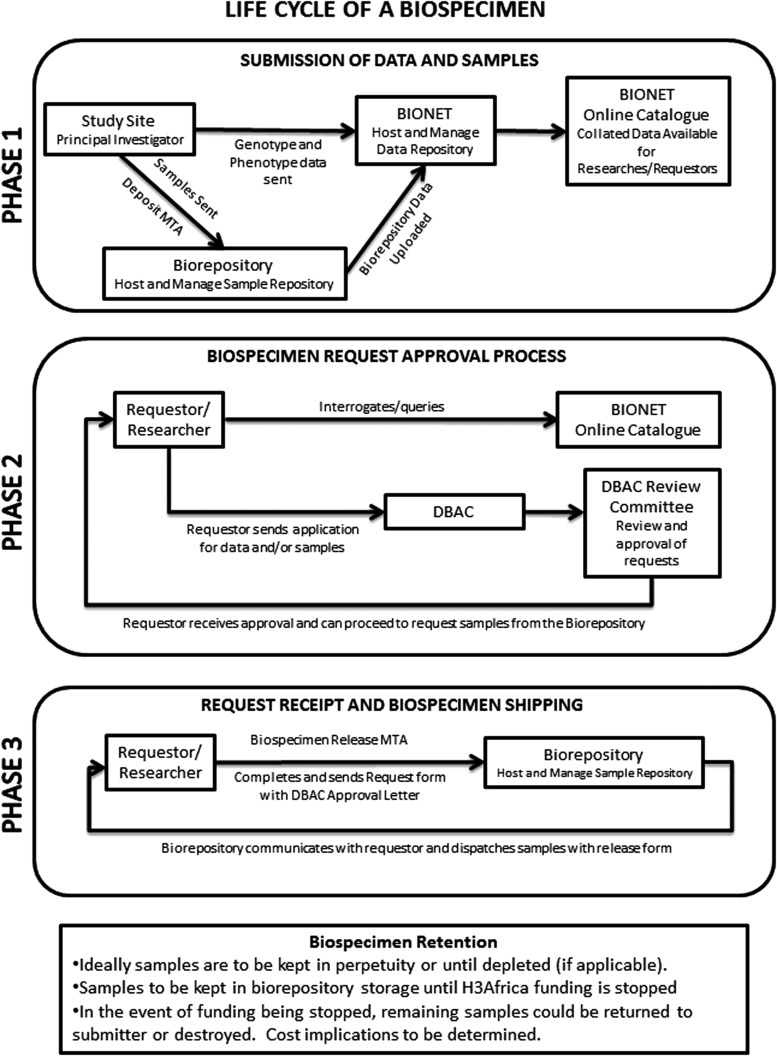
The life cycle of a biospecimen in the H3Africa Consortium includes the original submission of samples collected by H3Africa-funded investigators to a central biorepository for curation and storage. Phenotypic and genotypic data associated with each sample are hosted at the H3A BIONET and compiled into an online catalog (Phase 1). Non-H3Africa investigators who meet certain criteria can apply through a Data and Biospecimen Access Committee for access to the samples (Phase 2). If the request for samples is approved, the requestor and biorepository coordinate shipment of the samples by the optimal method (Phase 3).

Procedures for sample access were drafted to ensure optimal use of the limited amount of DNA available for each biospecimen. These procedures and the above policy elements were then integrated with information governing release of H3Africa genomic data sets to form the H3Africa Data and Biospecimen Access Guidelines. The guidelines were reviewed and approved by the H3Africa Steering Committee and posted on the consortium website (http://h3africa.org/consortium/documents). The process for requesting biospecimens from the H3Africa Consortium is illustrated in Figure 1.

Key points related to biospecimen access are presented here:

• Biospecimen requests must be approved by a DBAC comprising nine members with expertise in research relevant to H3Africa, biorepositories, data and informatics, African ethics, African legal matters, additional representatives from the health professions or the lay community. *Ad hoc* experts may be consulted as needed. Biospecimen requests will be accepted on a rolling basis with action taken within 60 days after the completed request is received.
• Only Senior Scientists and PIs are eligible to submit requests; PIs whose proposals are first undergoing peer review by a funding agency may ask the DBAC for a “preliminary letter of intent to share” for use in their grant applications.
• A Data and Biospecimen Access Request Form (to be made available on the H3Africa Consortium Website) must be completed, describing the proposed research question and methodology; evidence of funding and local ethics review; a description of the biospecimens requested; benefits of the research to Africa; the investigator's experience, expertise, and resources; and a plan for the disposal or transfer of unused biospecimens.
• Evaluation criteria are scientific merit of the request, conformity of the proposed research with the informed consent signed by the biospecimen donor and the donor's country laws, as well as the local ethics committee stipulations on biospecimen sharing, and compliance with H3Africa goals in specific biomedical research funding areas; potential for publication of the research and translation to biomedical practice; requestor's research expertise; and funding and resources.
• A commitment by the recipient scientist to return newly generated data from the biospecimens, e.g., additional genotyping and sequencing data, to H3ABioNet or EGA in order to make it widely accessible.

If the request for access to biospecimens is approved, the DBAC will send an approval letter to the requestor, who must then contact the host H3Africa biorepository to sign a Biospecimen Release Material Transfer Agreement and arrange for biospecimen shipment. Users must agree to furnish the DBAC with the citations for future publications resulting from shared resources. If a request is denied, the DBAC will correspond with the requestor regarding the reason for denial.

Achieving a fair, equitable, and efficient process for sharing of H3Africa biospecimens is expected to involve numerous challenges. One challenge is the limited amount of material from available biospecimens (mostly DNA isolated directly from whole blood). While whole-genome amplification has been discussed as the best way to provide an infinitely renewable DNA resource, the initial cost of such an approach will likely require the discovery of new funding sources. Therefore, the DBAC must balance the merit of the scientific question and the data that will be generated, and the appropriateness of the proposed methods with the remaining amount of sample to maximize the utility of resource.

Another challenge may involve differences in emerging biobanking policies across institutions and countries and over time. Ongoing tasks of the H3Africa Consortium will be to evaluate the effectiveness of the DBAC guidelines and implementation process, to track the use of biospecimens to evaluate whether meritorious science and the generation of valuable additional data are being facilitated, to maintain a feedback loop on the quality of the biospecimens, and to stay engaged in regional and international discussions around biobanking policy development, keeping an eye on maintaining policies and procedures that are balanced with the rights and intentions of stakeholders. Perhaps the greatest challenge to any biorepository is sustainability beyond the initial funding period. Solutions will require hard choices and innovative thinking by the H3Africa Consortium and biorepositories. Providing state-of-the-art, fee-based biorepository services to projects outside the H3Africa Consortium is critically important for long-term sustainability.

The H3Africa Consortium has developed a robust and comprehensive foundation to build genomic research capacity and excellence across the African continent. It is the intent of the biorepositories to contribute to this endeavor by providing genomic researchers with high-quality biospecimens that advance the goals of the H3Africa Consortium.
